# Effectiveness of Creatine Supplementation on Aging Muscle and Bone: Focus on Falls Prevention and Inflammation

**DOI:** 10.3390/jcm8040488

**Published:** 2019-04-11

**Authors:** Darren G. Candow, Scott C. Forbes, Philip D. Chilibeck, Stephen M. Cornish, Jose Antonio, Richard B. Kreider

**Affiliations:** 1 Faculty of Kinesiology and Health Studies, University of Regina, Regina, SK S4S 0A2, Canada; 2 Department of Physical Education, Brandon University, Brandon, MB R7A 6A9, Canada; ForbesS@BrandonU.CA; 3 College of Kinesiology, University of Saskatchewan, Saskatoon, SK S7N 5B2, Canada; phil.chilibeck@usask.ca; 4 Faculty of Kinesiology and Recreation Management, University of Manitoba, Winnipeg, MB R3T 2N2, Canada; Stephen.Cornish@umanitoba.ca; 5 Department of Health and Human Performance, Nova Southeastern University, Davie, FL 33314, USA; Exphys@aol.com; 6 Department of Health and Kinesiology, Texas A&M University, College Station, TX 77843-4253, USA; rbkreider@tamu.edu

**Keywords:** sarcopenia, dynapenia, mechanisms, exercise, functionality, safety

## Abstract

Sarcopenia, defined as the age-related decrease in muscle mass, strength and physical performance, is associated with reduced bone mass and elevated low-grade inflammation. From a healthy aging perspective, interventions which overcome sarcopenia are clinically relevant. Accumulating evidence suggests that exogenous creatine supplementation has the potential to increase aging muscle mass, muscle performance, and decrease the risk of falls and possibly attenuate inflammation and loss of bone mineral. Therefore, the purpose of this review is to: (1) summarize the effects of creatine supplementation, with and without resistance training, in aging adults and discuss possible mechanisms of action, (2) examine the effects of creatine on bone biology and risk of falls, (3) evaluate the potential anti-inflammatory effects of creatine and (4) determine the safety of creatine supplementation in aging adults.

## 1. Introduction

In 2016, the World Health Organization established an International Classification of Disease, 10th Revision, Clinical Modification (ICD-10-CM; M62.84) code for sarcopenia as a means for better diagnosis, assessment and treatment of the disease [1]. The European Working Group on Sarcopenia in Older People recently defined sarcopenia as a disease characterized by low muscle strength, muscle quantity/quality and physical performance [2]. Sarcopenia typically occurs in 6–22% of adults ≥65 years of age [3] and is associated with reduced bone mass [4] and elevated low-grade inflammation (i.e., “inflamm-aging”; [5]). The age-related loss in bone mass and bone strength (i.e., osteoporosis) increases bone fragility and, along with an increased risk for falls, this increases risk of subsequent fracture [6]. Inflamm-aging has a negative effect on muscle protein metabolism and satellite cell function [5], two main contributing factors of sarcopenia (for a comprehensive and detailed review on the mechanisms of sarcopenia, please see [7]). The age-related loss in muscle mass is a small contributing factor to the decrease in muscle strength with aging, referred to as dynapenia [8,9]. Dynapenia leads to premature morbidity and mortality [10]. Therefore, interventions which have beneficial effects on aging muscle mass, strength (dynapenia), bone, falls prevention and inflammation are warranted. 

Accumulating evidence (meta-analyses, randomized controlled trials) suggests that creatine supplementation has the potential to increase aging muscle mass and muscle strength, reduce the risk of falls and perhaps attenuate the loss of bone mineral. Preliminary evidence also indicates that creatine may have anti-inflammatory properties. The purpose of this review is to: (1) summarize the effects of creatine supplementation, with and without resistance training, in aging adults and discuss possible mechanisms of action, (2) examine the potential effects of creatine on bone biology and risk of falls, (3) evaluate the potential anti-inflammatory effects of creatine and (4) determine the safety of creatine supplementation in aging adults. 

## 2. Creatine Synthesis and Metabolism

**Figure 1:**
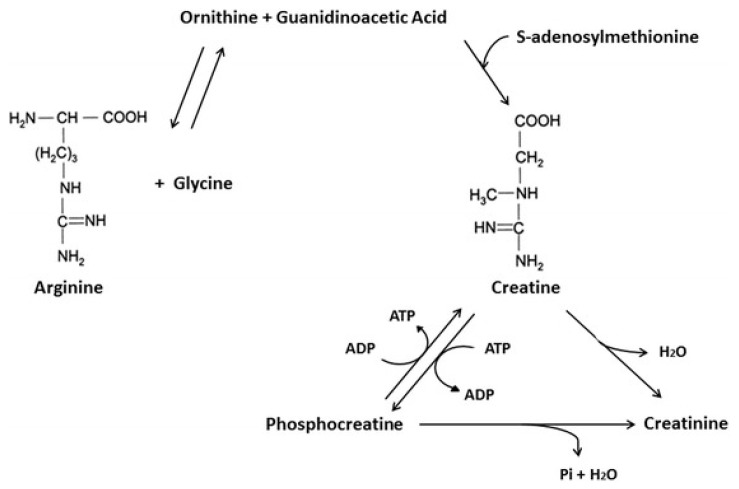
Endogenous creatine synthesis. Adopted from Kreider and Jung [14]. ATP = Adenosine Triphosphate. ADP = Adenosine Diphosphate.

Creatine is a nitrogenous organic acid found primarily in red meat, seafood [11] and poultry [12]. The vast majority of creatine resides in skeletal muscle (intramuscular; ~95%) with trivial amounts located in the testes and brain (~5%) [13,14]. About 66% of intramuscular creatine is phosphocreatine (PCr), with the remaining being free creatine. Creatine is endogenously produced from reactions involving the amino acids arginine, glycine and methionine in the kidney and liver [11] (Figure 1). Approximately 1–2% of intramuscular creatine is degraded daily to creatinine (metabolic by-product) and excreted in the urine [15,16,17]. Therefore, 1–3 grams/day of creatine is required to maintain normal creatine levels. About 50% of this daily requirement is provided by dietary sources (i.e., red meat, seafood, poultry) [12,18], with the remaining contribution coming from endogenous creatine synthesis.

The primary metabolic role of creatine is to combine with inorganic phosphate (Pi) to form PCr through the enzymatic reaction involving creatine kinase (CK). As adenosine triphosphate (ATP) is degraded into adenosine diphosphate (ADP) and Pi to provide free energy for metabolic activity (i.e., exercise), the free energy released from the hydrolysis of PCr into Cr + Pi can be used as a buffer to resynthesize ATP [19,20]. 

## 3. Creatine and Aging Muscle

Dynapenia is an indicator of sarcopenia [2]. Muscle strength remains relatively constant until the fifth decade of life and then begins to decline at a rate of 1.2–1.5% per year thereafter [21]. Similarly, muscle mass begins to decline at a rate of ~0.8% per year after the age of 50 [22]. Evidence is accumulating that creatine supplementation can have a positive effect on aging muscle. This section reviews the effects of creatine supplementation, with and without resistance training, in aging adults. We have previously reviewed individual studies examining the effects of creatine supplementation during resistance training in aging adults [23,24,25,26]. Therefore, only meta-analyses on creatine supplementation during resistance training are summarized. 

### 3.1. Creatine Supplementation During Resistance Training: Summary of Meta-Analyses

In the most recent meta-analysis, Chilibeck et al. [27] showed a significantly greater increase in lean tissue mass (1.37 kg) and upper- and lower-body maximal strength in aging adults (*n* = 721; 57–70 years of age) who supplemented with creatine during resistance training (7–52 weeks) compared to placebo during resistance training. Devries and Phillips [28] also showed that creatine supplementation during resistance training (7–26 weeks) increased lean tissue mass (1.33 kg), upper- and lower-body strength and physical performance (3 s chair stand test) in aging adults (*n* = 357; 55–71 years of age) compared to placebo. The greater increase in lower-body strength from creatine [27,28] is especially important as lower-body muscle groups are more negatively affected during the aging process [29]. Finally, Candow et al. [30] determined that creatine supplementation during resistance training (>6 weeks) in over 300 aging adults increased lean tissue mass (0.94 kg) and chest press strength more than placebo and resistance training. Collectively, creatine supplementation during resistance training appears to be an effective intervention for increasing aging muscle mass, strength and functional physical performance. 

### 3.2. Creatine Supplementation without Resistance Training

Results showing a beneficial effect on aging muscle from creatine supplementation without concomitant resistance training are limited. Four studies have shown positive effects, three studies have shown no effect, and one study has shown mixed effects. Stout et al. [31] found a significant improvement in hand-grip maximal strength in aging adults who supplemented with creatine (20 g/day for 7 days followed by 10 g/day for 7 days) compared to placebo. Furthermore, Rawson et al. [32] showed that 1 month of creatine supplementation (20 g/day for 10 days followed by 4 g/day for 20 days) reduced lower body muscle fatigue in aging males (60–82 years of age). Gotshalk et al. [33,34] found an increase in strength and functionality in aging adults who supplemented with creatine (0.3 g/kg/day) for one week. In contrast to these findings, Chami and Candow [35] found no effect from creatine supplementation (0.1–0.3 g/kg) for 10 days on muscle strength, endurance or physical performance in aging adults. Very-low-dosage creatine (1 g/day) ingestion for 1 year also failed to produce muscle benefits in aging postmenopausal women [36]. Finally, an acute bolus ingestion of creatine (20 g) had no effect on muscle strength or endurance in aging males [37]. In aging females (*n* = 10; 67 ± 6 years of age), 7 days of creatine supplementation (0.3 g/kg/day) significantly improved lower-extremity physical performance (sit-to-stand test) but failed to improve indices of endurance capacity [38]. Results across studies provide equivocal evidence that creatine supplementation, independent of resistance training, improves some measures of muscle and physical performance in aging adults.

### 3.3. Possible Cellular Mechanisms of Creatine

Creatine supplementation may work through several cellular mechanisms to enhance muscle mass and muscle/physical performance. Safdar et al. [39] assessed a global array of mRNAs and proteins following 10 days of creatine supplementation (20 g/day for 3 days followed by 5 g/day for 7 days) in young men. Creatine supplementation upregulated proteins involved in osmolarity. Osmolarity-induced cellular swelling may increase the expression of myogenic transcription factors which stimulate satellite cell activity [39]. Creatine supplementation during 12 weeks of resistance training in young males significantly increased myogenic transcription factor protein expression of myogenin and MRF-4 [40]. Creatine has also been shown to increase the expression of protein kinases downstream in the mammalian target of the rapamycin (mTOR) muscle protein synthetic pathway [39]. Furthermore, insulin-like growth factor-1 (IGF-1) activates the mTOR signaling pathway and creatine supplementation in young males has been shown to increase IGF-1 mRNA expression and the phosphorylation of 4E-BP1 after an acute bout of resistance exercise [41]. From an anti-catabolic perspective, creatine supplementation decreases leucine oxidation (an indicator of muscle protein catabolism) in young adults [42] and urinary 3-methylhistidine (an indicator of whole-body protein catabolism) in aging adults [43,44]. The anti-catabolic effect of creatine may be due to its potential as an antioxidant [45]. With aging, there is damage to mitochondria, most likely caused by mutations in mitochondrial DNA, resulting in deficits in the respiratory chain [46]. Altered respiratory chain function results in the generation of reactive oxygen species, which have been implicated in the damage of cellular membranes and inflammation, leading to muscle damage and proteolysis [46]. Creatine may protect against oxidative stress to mitochondria. For example, in mouse myoblast cells that were subjected to oxidative damage (through H_2_O_2_ intoxication), supplementation with creatine attenuated the reduction in differentiation and reduced signs of mitochondrial damage as assessed by electron microscopy [47]. Creatine therefore appears to protect against mitochondrial damage caused by oxidation and this may translate to reduced inflammation and muscle damage with aging. Recent work in humans indicated that providing a supplement that contains creatine (i.e., 2.5 g/day) during 12 weeks of resistance and high-intensity interval training reduced markers of inflammation [48].

## 4. Creatine and Aging Bone

Prevention of bone fracture in older adults is dependent on two factors: (1) the strength of bone and (2) the prevention of falls [49]. This section reviews the potential for creatine supplementation, especially when combined with resistance training, for increasing bone strength and reducing the risk of falls in aging adults. There are a number of studies indicating a positive effect of creatine in young (growing) animal models or children and young adults for either enhancing bone mineral properties or decreasing bone resorption [50,51,52,53]. We have previously reviewed these studies in detail [23]. Therefore, the focus of this review is on aging animal models and older adults only.

### 4.1. Creatine and Bone: Cellular Studies

The amount of creatine in bone relative to other tissues is quite small—95% of creatine is found in muscle, with most remaining stores in the brain, liver, kidney and testes [54]. Therefore, bone would account for less than 5% of the body’s creatine stores. Cells involved in bone formation (i.e., osteoblasts) require large amounts of energy during the deposition of bone matrix and mineralization; this energy requirement decreases drastically when osteoblasts complete their role of bone formation during the bone remodeling cycle and transform into osteocytes (i.e., inactive bone lining cells) [55]. The requirement for PCr breakdown to buffer ATP levels in bone is therefore most likely highest when osteoblasts are active during the bone remodeling cycle. Indeed, PCr is not detectable in the transition zone of mineralized cartilage and bone (i.e., the transition zone between immature bone (osteoid) and mineralized bone [56]). There is evidence that creatine or CK is important for the activation of osteoblasts (i.e., cells involved in bone formation). Compounds that stimulate bone formation, such as vitamin D and insulin-like growth factor-1, increase the activity of CK in rat osteoblasts when added to cell cultures [57,58]. When creatine is added to osteoblast cells in vitro, the differentiation and activation of osteoblasts are enhanced [59]. On the other hand, CK activity may also be important for the activation of osteoclasts (i.e., cells involved in bone resorption). Pharmacological suppression of CK in vitro suppresses bone resorption by osteoclasts, and mice that were genetically modified to be missing the gene for CK were protected against bone loss induced by ovariectomy (i.e., an animal model of menopause) [60]. Cellular studies are therefore unclear regarding the effects of creatine, as the CK reaction seems important for cells implicated in both bone formation and resorption.

### 4.2. Effect of Creatine on Properties of Bone (Animal Studies)

Studies involving ovariectomized rats or male mice using Fourier-transform (FT)-Raman spectroscopy, an optical technique for assessing bone mineral content, have suggested a positive effect of creatine supplementation on bone [61,62]. Ovariectomized rats that were supplemented with 300 mg/kg/day creatine (which equates to about 21 g/day in humans) for 8 weeks had a significant increase in phosphate content in the trabecular bone of the lumbar vertebrae [62] and male mice who were supplemented with either 0.5 g or 2 g/kg/day creatine for 30 days had a decrease in the carbonate/phosphate ratio assessed in the femur [61]. The effect was not different between doses of creatine. An increase in bone phosphate content or a decrease in carbonate/phosphate ratio is associated with lower fracture risk in aging women [63]; therefore, these changes in bone with creatine supplementation are implied to have a protective effect against fracture.

In contrast to these studies, the evaluation of bone using techniques aside from FT-Raman spectroscopy has failed to show positive effects for preventing bone loss in aging animal models. Nine weeks of creatine supplementation (5 g/kg/day) in spontaneously hypertensive rats (an experimental model of osteoporosis) failed to affect femoral or lumbar spine bone mineral density, as assessed by dual energy X-ray absorptiometry [64]. It is proposed that creatine supplementation may be most effective if combined with resistance training because creatine increases muscle mass [27] in aging adults and the increased muscle mass may allow greater pull on bone during muscle contraction, which would increase bone strain and induce mechanotransduction to bone cells to increase bone formation [65]. The only animal study to combine creatine supplementation with exercise training, however, did not demonstrate a positive effect of creatine on bone [66]. Ovariectomized rats were trained by downhill treadmill running (which induces a heavy eccentric overload on muscle) or combined treadmill training with 300 mg/kg/day creatine supplementation. Compared to the control, exercise training was effective for improving most bone measures; however, the addition of creatine had no further benefit on bone mineral density at the femur, lumbar spine, or whole body; no effect on femoral strength assessed through three-point bending (this included maximal load, stiffness and toughness) or bone histomorphometric properties from the femur, including measures such as trabecular number, thickness and spacing, as well as measures of bone formation and resorption [66]. Overall, it appears that the effect of creatine supplementation on the bone health of animal models of aging is equivocal.

### 4.3. Effect of Creatine on Properties of Bone (Human Studies)

Aging males (55–77 years) who were supplemented with creatine (0.1 g/kg/day) during a 10 week supervised resistance training program (3 days/week) had a 27% reduction in a marker of bone resorption (i.e., urinary n-telopeptide cross-links of type 1 collagen) compared to a 13% increase in participants who took placebo during resistance training [43]. As mentioned in the section on cellular studies, creatine is capable of stimulating the activation and differentiation of osteoblasts (cells involved in bone formation) [59]. The stimulation of osteoblasts causes the release of a hormone-like protein (osteoprotegerin) which inhibits the activation of osteoclasts (cells involved in bone resorption) [67]. In this manner, the stimulation of osteoblasts by creatine supplementation [59] may downregulate the activation of osteoclasts to reduce bone resorption [43]. Despite this potential promising effect of creatine supplementation, additional studies that evaluated creatine supplementation (5 g/day) for 14–26 weeks in aging adults during resistance training programs have failed to detect an impact on blood or urine markers of bone formation or resorption [68,69,70]. One additional long-term study (12 months), with very low-dosage creatine (1 g/day) and without resistance training, in osteopenic postmenopausal women also failed to show an impact from creatine on markers of bone resorption or formation [36]. 

As with its effects on markers of bone turnover, the effect of creatine on bone mineral properties in aging adults is equivocal. A recent meta-analysis [71] assessed five studies regarding the effectiveness of creatine on bone [65,69,70,72,73]. These studies assessed 3–12 months of creatine supplementation (5–8 g/day) with a loading phase of about 20 g/day for 5 days in two of the studies [65,69], combined with resistance training (2–3 days/week) in males and females (57–85 years of age). Results of the meta-analysis showed no greater effect from creatine supplementation on bone mineral density at the total hip, femoral neck, lumbar spine or whole body [71]. One additional study, published after this meta-analysis, also showed no greater effect from creatine supplementation (~8 g/day) combined with resistance training (3 times per week for 8 months) versus placebo on lumbar spine, hip, femoral neck or whole-body bone mineral density in aging males and females (mean age 55 years) [74]. When the results of this study were added to the meta-analysis by Forbes et al. [71], the results remained unchanged (i.e., no significant effects of creatine supplementation were found on any bone site). It should be noted that bone takes a long time to turn over and therefore longer interventions are more likely to be effective. The most effective intervention showed that creatine supplementation (~8 g/day) over 12 months combined with resistance training (3 times per week) in postmenopausal women attenuated the loss of bone mineral density at the femoral neck compared to placebo (i.e., loss of 1.2% in the creatine group versus 3.9% in the placebo group) [72]. This study also showed that geometric properties at the femur (i.e., femoral shaft subperiosteal width) were increased with creatine supplementation; this may positively influence the bending strength of bone. Limitations of this study included a relatively small sample size and the fact that the bone mineral density loss in the placebo group was quite high, leading to the possibility that the findings were by chance. Studies of greater length that include measures that predict bone strength (e.g., bone geometric properties) aside from bone mineral density are needed to determine for certain whether creatine supplementation can affect bone mineral during resistance training in aging adults. One recent 12 month study involving a relatively large number of osteopenic postmenopausal women (*n* = 108) found no effect from creatine (1 g/day) on bone mineral density (i.e., lumbar spine, proximal femur) or bone micro-architectural properties assessed at the distal radius and tibia with high-resolution peripheral quantitative computed tomography (i.e., trabecular number, thickness and spacing) [36]. The strength of this study was the use of more advanced imaging techniques to evaluate bone; however, the dose of creatine was very small (at least five times smaller than doses used in other studies) and no resistance training intervention was used. As mentioned earlier, creatine supplementation is effective for increasing muscle mass in aging adults [27] and one of the putative mechanisms by which creatine might positively affect bone is the increased strain on bone that a greater muscle mass (and therefore strength) would allow when performing muscle contractions [65].

### 4.4. Effect of Creatine on Reducing the Risk of Falls

**Figure 2:**
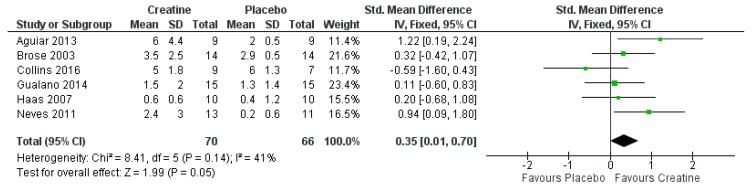
Forest plot for sit-to-stand performance. SD = Standard Deviation; CI = Confidence Interval. The large diamond on the Forest plot indicates the mean effect across studies. The effect is significant if the diamond does not cross the “zero” point on the x-axis.

Along with weaker bones, aging adults are at an increased risk of falling because of reduced muscle mass and loss of motor coordination, which decreases stability and reduces ability to maintain balance [49]. A study involving aging mice found that long-term creatine supplementation improved the expression of genes involved in neuronal growth and neuroprotection, which led to a trend of significant improvement in motor function, as measured by increased locomotor activity (*p* = 0.054) [75]. Translated to aging humans, this increased motor coordination could be effective for improving balance and preventing falls. A previous meta-analysis (three studies) found a significant improvement in a sit-to-stand test (i.e., ability to perform a given number of repetitions of repeated rising and sitting in a chair over a given amount of time, or ability to perform a set amount of chair rises in the fastest time) in aging males and females supplementing with creatine versus placebo during resistance training [28]. We added three additional studies published since this original meta-analysis and ran a new meta-analysis, using methods previously described [27,71]. The included studies involved aging males and females (mean ages of 57–69 years across studies) randomized to receive creatine (5 g/day; with three studies including a loading phase of 20 g/day for the first 5 days) or placebo and participating in resistance training (2–3 times per week) for 12–24 weeks [68,69,76,77,78,79]. This new meta-analysis indicated that creatine supplementation results in a significant improvement in sit-to-stand performance compared to placebo (*p* = 0.05; Figure 2). This is equivalent to a 23% improvement in the creatine-supplemented participants compared to a 16% improvement in placebo-supplemented participants. This novel finding has clinical significance because the sit-to-stand test is a good predictor of reduced risk for falls in aging adults [80]. Our previous meta-analysis involving aging males and females randomized to receive creatine or placebo during resistance training programs showed that creatine significantly improved leg strength (*p* = 0.01) [27]. This again is clinically relevant, as leg strength is important for stability and is another good predictor of reduced risk of falls in older adults [80]. An additional commonly-used test for risk of falls is the timed up-and-go test, which involves getting up from a chair, walking a set distance (usually 3 m), and turning around and returning to a sitting position [81]. This has been used in a couple of studies involving creatine supplementation combined with resistance training; however, no significant improvements (over placebo) were found from creatine [69,77]. No study has actually evaluated the number of falls from creatine and resistance training. This would require an intervention with a long-term follow-up in a large cohort of individuals. Overall, it appears that creatine supplementation during resistance training programs in aging adults is effective for improving some risk factors for falling.

## 5. Potential Anti-Inflammatory Effects of Creatine Supplementation 

Elevated and sustained low-grade inflammation during the aging process (i.e., inflamm-aging) has a negative effect on aging muscle [82] and bone [83]. Creatine has been shown to act as an antioxidant [45,84] and, as such, may reduce indices of inflammation in aging adults. There is a strong link between inflammation and the production of reactive oxygen species by neutrophils to degrade and digest foreign or damaged tissue [85]. Elevated age-related inflammation and oxidative stress decreases muscle protein synthesis [86]. Potentially, creatine supplementation may play a role in downregulating oxidative stress associated with inflammation. This section reviews and summarizes the possible anti-inflammatory effects of creatine supplementation.

### 5.1. Creatine and Cell/Animal Studies

In an in vitro study, creatine (0.5–5 mM) was able to suppress the adhesion of neutrophils to endothelial cells and inhibit the binding of intracellular adhesion molecule-1 (ICAM-1) and E-selectin, suggesting an anti-inflammatory effect form creatine [87]. In a rat model of lung ischemia/reperfusion, creatine supplementation was able to reduce acute lung injury through anti-inflammatory mechanisms which included an attenuation of Toll-like receptor 4 (TLR-4; a signal for the activation of nuclear factor kappa B (NF-κB) and the initiation of the innate immune system inflammatory response) [88]. Long-term creatine supplementation (1 year) in Sprague-Dawley rats had no negative effect on liver histology [89]. Collectively, these preliminary results indicate that creatine supplementation has the potential to decease markers of inflammation. 

### 5.2. Creatine and Exercise

There have been several studies that have evaluated the effects of creatine supplementation on a variety of systemic inflammatory mediators after an acute bout of exercise. Santos et al. [90] showed that pre-exercise creatine loading (20 g/day) in marathon runners attenuated the rise in prostaglandin-E_2_ (PGE_2_), tumor necrosis factor-alpha (TNF-α) and serum levels of creatine kinase (indicator of muscle damage) compared to placebo. Using the same creatine supplementation protocol, Bassit et al. [91] observed a significantly smaller increase in TNF-α, interferon-α (IFN-α), interleukin-1β (IL-1β) and PGE_2_ post-exercise compared to placebo in elite athletes who completed a half-ironman. Deminice et al. [92] showed that creatine supplementation (0.3 g∙kg^−1^) for 1 week blunted the rise in TNF-α and C-reactive protein (CRP) in young soccer players compared to placebo but had no effect on indicators of oxidative stress. In addition, Deminice and Jordao [93] showed that creatine supplementation (2% diet) for 28 days prior to performing 1 h of swimming decreased oxidative stress in Wistar rats. In contrast to these positive findings from creatine, Rawson et al. [94] showed no effect from 10 days of creatine supplementation on serum creatine kinase levels in resistance-trained males who performed an intense lower-body training session. Although speculative, creatine supplementation may provide anti-inflammatory effects for aerobic-type but not resistance-type activities. Future research should directly compare the anti-inflammatory effects of creatine in aerobic- vs. resistance-type activities. 

Given the potential of creatine supplementation to reduce inflammation, Cornish and Peeler [95] recently examined the effects of a 12-week creatine (20 grams/day for 1 week and then 5 grams/day for 11 weeks) versus placebo supplementation protocol on inflammatory biomarkers (C-reactive protein, interleukin-1β, interleukin-6, s100 A8/A9, and tumor necrosis factor-α) in individuals diagnosed with knee osteoarthritis [95], a condition characterized by elevated low-grade inflammation. Compared to placebo, creatine had no effect on any marker of inflammation. However, heart failure patients who consumed creatine (5 g∙day^−1^) and performed aerobic exercise (3 days∙week^−1^) experienced a significant reduction in systemic IL-6, CRP, and endothelial function (ICAM-1, P-selectin) compared to control patients [96].

In summary, research demonstrating an anti-inflammatory effect from creatine supplementation is limited. Creatine may attenuate the increase in a pro-inflammatory immune system response to aerobic exercise, but appears to provide no inflammatory benefit for resistance-trained or osteoarthritic individuals.

## 6. Practical Exercise Recommendations

Given the potential benefits of creatine supplementation in combination with exercise interventions (primarily resistance exercise) compared to exercise alone [27], it is prudent to provide practical recommendations. The Canadian Society for Exercise Physiology and the American College of Sports Medicine [97] provide similar exercise guidelines for older adults, consisting of 150 min per week of moderate to vigorous intensity aerobic activity and resistance training at least 2 days per week consisting of 8–10 exercises of 8–12 repetitions. In young adults, when training volume was equated, resistance training 2 or 3 days per week while supplementing with creatine only on training days led to greater improvements in muscle thickness compared to resistance training alone [98]. There are several training methods that may be augmented by creatine supplementation such as drop set training [44] or whole-body multi-set training [99]. For example, Candow et al. [99] had participants follow a supervised whole-body resistance training program for 32 weeks. Participants first performed 5 min of light-intensity cycling to warm-up. Participants then complete three sets of 10 repetitions (~75% one-repetition maximum) with 1–2 min rest between sets for 11 exercises (leg press, chest press, lat pull-down, shoulder press, leg extension, leg curl, triceps extension, biceps curl, calf press, back extension and abdominal curl). To maintain progressive overload, once participants were able to complete three sets of 10 without muscular fatigue, resistance was increased 2–10 kg. It is important to note that any exercise intervention needs to be individualized. For example, programs may differ based on training goals, past training experience, and current training status. Furthermore, it is recommended that training programs be designed and supervised by a qualified exercise professional.

## 7. Safety of Creatine for Aging Adults

Direct evaluation of the safety of creatine supplementation in aging adults is limited. However, creatine does not appear to jeopardize liver or kidney function or lead to cytotoxicity. For example, in frail aging adults (*n* = 9, 70 ± 5 years), the addition of creatine supplementation (5 g/day) to protein during 14 weeks of resistance training had no effect on blood urea, creatinine, bilirubin, alkaline phosphatase, gamma glutamyltransferase, alanine aminotransferase, aspartate aminotransferase or creatine kinase [77]. In postmenopausal women (*n* = 23; 57 ± 6 years of age), creatine supplementation (0.1 g/kg/day) for 1 year had no effect on markers of liver (bilirubin, aspartate aminotransferase, alanine aminotransferase and alkaline phosphatase) or kidney (urea, albumin, microalbumin, urine protein and creatinine clearance) function [72]. Lobo et al. [36] also found no detrimental effect from 1 year of creatine (1 g/day) on urinary albumin in postmenopausal women (*n* = 56). In aging patients (*n* = 13; >45 years of age) with type II diabetes, a disease associated with sarcopenia [100], creatine supplementation (5 g/day) for 12 weeks had no effect on albuminuria, proteinuria, albumin:creatinine ratio, urea and creatinine and estimated creatinine clearance compared to placebo [101]. Furthermore, aging males (*n* = 23, 59–77 years of age) who supplemented with creatine (0.1 g/kg/day) over 10 weeks of supervised whole-body resistance training experienced no difference in urinary formaldehyde production compared to placebo, suggesting that creatine does not result in cytotoxicity [43]. Patients with Parkinson disease (*n* = 40; 60 ± 9.4 years of age) who ingested creatine for 2 years (20 g/day for 6 days followed by 2 g/day for 6 months followed by 4 g/day for the remainder of the study) experienced no increase in markers of renal dysfunction (tubular damage, glomerular filtration rate, microalbumin, hematuria, renal damage) compared to placebo [102]. A recent position stand paper by the International Society for Sports Nutrition concluded that creatine is a safe intervention for aging adults [103].

## 8. Summary and Conclusion

Sarcopenia is a multifactorial disease characterized by a progressive reduction in muscle mass, strength (dynapenia) and physical performance. The etiology of sarcopenia is complex and involves changes in muscle fiber morphology, neuromuscular activity, protein kinetics, endocrinology and inflammation. Sarcopenia is associated with reduced bone mass and bone strength and may be a contributing factor for the increased risks of falls and fractures often observed in aging adults. It is well established that resistance training is an effective lifestyle intervention for improving aging muscle mass, strength and bone accretion. Accumulating evidence indicates that creatine supplementation, with and without resistance training, has possible anti-sarcopenic and anti-dynapenic effects. Specifically, creatine supplementation increases aging muscle mass and strength (upper- and lower-body), possibly by influencing high-energy phosphate metabolism, muscle protein kinetics and growth factors. Creatine supplementation has shown potential to enhance bone mineral in some but not all studies, and seems to affect the activation of cells involved in both bone formation and resorption. Creatine has the potential to decrease the risk of falls experienced by aging adults which would subsequently reduce the risk of fracture. Finally, preliminary evidence suggests that creatine may have anti-inflammatory effects during times of elevated metabolic stress, such as during extended/intense aerobic exercise. Creatine does not appear to reduce indicators of inflammation during resistance training. Although research is limited, creatine supplementation does not appear to negatively affect markers of liver or kidney function in aging adults. Future research should objectively examine the safety and long-term effects of creatine supplementation on properties of muscle, bone and inflammation in various aging and disease-state populations.
